# Giant intermuscular lipoma of breast: A case report

**DOI:** 10.1016/j.jpra.2023.10.008

**Published:** 2023-10-14

**Authors:** Kristupas A. Suslavičius, Daiva Gudavičienė, Nerijus Jakutis

**Affiliations:** a Faculty of Medicine, Medical Academy, Lithuanian University of Health Sciences, Kaunas, Lithuania; b Department of Plastic and Reconstructive Surgery, Vilnius University Hospital Santaros Klinikos, Vilnius, Lithuania; c Clinic of Rheumatology, Orthopaedics Traumatology and Reconstructive Surgery, Institute of Clinical Medicine, Faculty of Medicine, Vilnius University, Vilnius, Lithuania

**Keywords:** Breast, Intermuscular lipoma, Giant lipoma, Pectoral muscles, Soft tissue tumor, Surgical excision

## Abstract

A giant intermuscular lipoma, an exceedingly rare occurrence, constitutes a non-malignant neoplasm originating from the mesodermal germ cell layer, with dimensions surpassing 10 cm. Its differentiation from liposarcoma and other malignant tumours is imperative. We present a case involving a 75-year-old woman who initially raised suspicions of liposarcoma due to pronounced enlargement and fullness in the upper quadrants of the left breast. After comprehensive imaging evaluations, the identification of a sizable BI-RADS 4a lesion positioned between the major and minor pectoral muscles of the left breast was found. The definitive diagnosis of an exceedingly rare giant intramuscular lipoma was validated solely subsequent to the surgical excision of the lipoma, through histological analysis.

## Introduction

Lipomas are defined as non-malignant neoplasms that arise from the mesodermal germ cell layer, which most commonly occur in specific anatomical regions, including the thigh, shoulder, and trunk.^1^ Lipomas can be categorized into different types, including superficial lipomas, deep lipomas, intramuscular or intermuscular lipomas, and osteolipomas. Although lipomas are relatively common, affecting ∼1 % of the population, intermuscular lipomas are rare, representing only 0.3 % of all lipomas.^2^

Here we present a rare case of a giant intermuscular lipoma (IL) situated between the major and minor pectoral muscles.

## Case report

**Figure 1:**
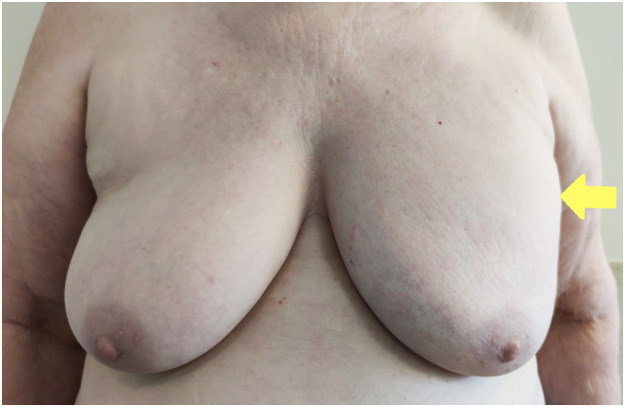
Preoperative frontal view of the breasts. The yellow arrow reveals marked enlargement and fullness in the upper quadrants of the left breast.

A 75-year-old woman with a 3-month history of discomfort due to a lump in the left breast was admitted to the Department of Plastic and Reconstructive Surgery at Vilnius University Hospital. The patient underwent thorough examination at the National Cancer Institute, revealing breast asymmetry, with pronounced enlargement and fullness in the upper quadrants of the left breast (Figure 1). Palpation of the left breast evoked tenderness, although the nipple architecture was unchanged. The patient had no history of trauma or injury and no family history of breast cancer or other oncological pathologies. However, she had a history of surgical interventions, including excision of a melanoma from the right calf, thyroid gland surgery, and multiple procedures for ectopic pregnancy. Hypothyroidism, recurrent tachycardia, and pulmonary arterial hypertension have been previously diagnosed, and the patient was already undergoing a therapeutic regimen of l-Thyroxine, Nebilet, and Triplixam. The blood tests conducted fell within the normal range.

**Figure 2:**
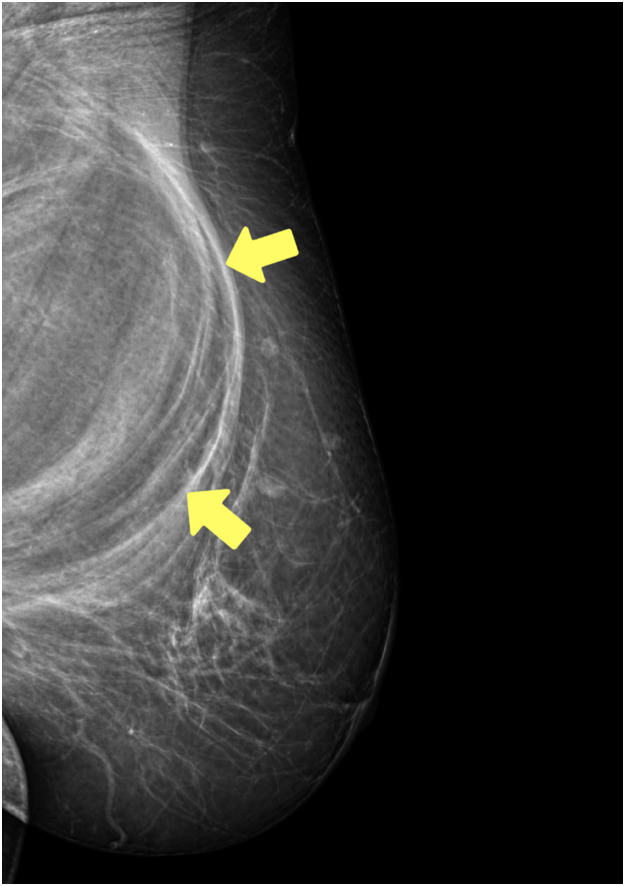
Preoperative mammography. The yellow arrow shows a partially encompassing fatty-density structure of approximately 13 cm along the chest wall on the left. In the upper outer quadrant of the left breast, three intramammary lymph nodes are identified.

Mammography revealed a heterogeneous tissue composition within the breasts. No discrete lesions or microcalcifications were observed on the right side; however, a cluster was observed adjacent to the nipple. On the left, a partially encompassing fatty density structure of ∼13 cm was noted along the chest wall. Three intramammary lymph nodes were identified in the upper-outer quadrant of the left breast. The skin and nipples exhibited no discernible alterations (Figure 2).

Ultrasonography (US) revealed a substantial lipoma-like structure, measuring ∼13 cm in the lateral left breast. Adjacent to and slightly above the nipple, an area of mixed echogenicity spanning ∼3.5 cm was observed. Normal lymph nodes were up to 1.5 cm long in the left axilla. Conversely, the right breast displayed no discernible formations and the right axilla contained no anomalous lymph nodes.

**Figure 3:**
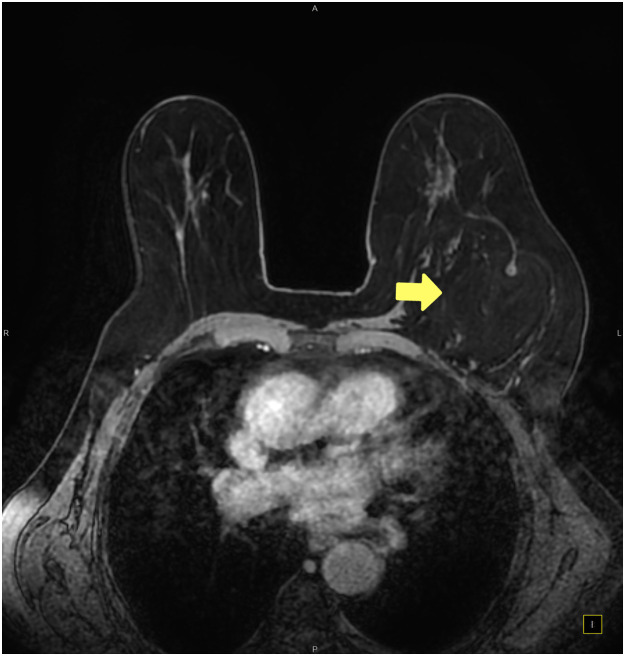
Preoperative MRI. The yellow arrow shows a formation between major and minor pectoral muscles measuring approximately 60 × 80 × 130 mm in the left breast. Notably, contrast-enhancing foci, reminiscent of parenchymal tissue, are observable bilaterally across the breasts.

Magnetic resonance imaging (MRI) revealed a mass between the major and minor pectoral muscles, measuring approximately 6 × 8 × 13 cm in the left breast, and contrast-enhancing foci reminiscent of parenchymal tissue were observed bilaterally across the breasts. No enlarged lymph nodes were identified in the axillary regions (Figure 3). Further assessment using US-guided Breast Imaging-Reporting and Data System categorisation defined the right breast as category 1, indicating the absence of malignancy.^3^ However, the left breast was 4a, indicating a 2–10 % probability of malignancy. Hence, surgical biopsy was required to definitively ascertain the presence or absence of breast cancer.

**Figure 4:**
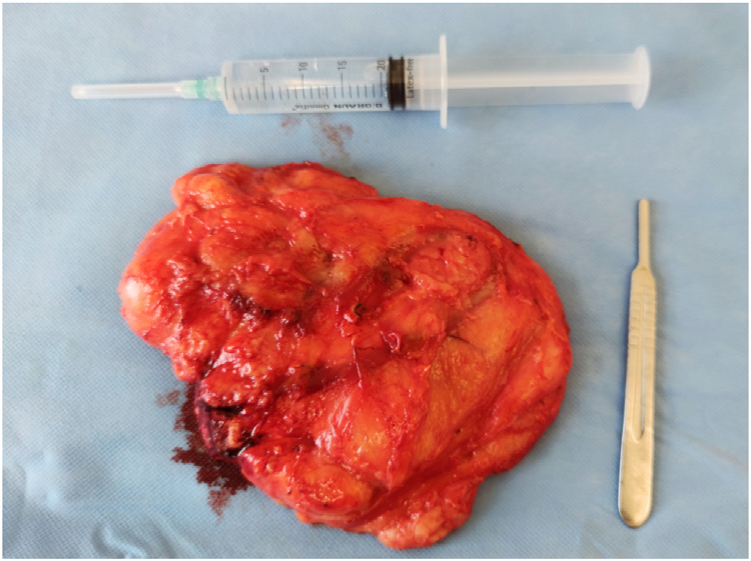
22 × 11 cm intermuscular lipoma.

The patient underwent surgical intervention under general anaesthesia. The lateral border of the major pectoral muscle was accessed and tracked along its contour. Intraoperatively, an elevated major pectoral muscle was observed, with a subjacent soft mass. A 22 × 11-cm soft yellowish lipoma was carefully excised (Figure 4) by dissection following the trajectory of the major pectoral muscle toward the sternum, with sharp separation of the medial edge. Histological analysis of the excised mass confirmed the diagnosis of an IL. Postoperatively, the patient recovered without complications, facilitated by administration of low-molecular-weight heparin and diclofenac. No indications of recurrence were observed at follow-up 1 year postoperatively.

## Discussion

An IL arises from the infiltration of undifferentiated, fully mature adipocytes within the interstitial spaces of muscle fibres and myocytes. The precise aetiology of lipomas remains elusive, although one hypothesis posits that the coalescence of endocrine, metabolic, and genetic aberrations could potentially underlie the exaggerated proliferation of mature adipocytes.^4^ One study found that an IL predominantly affects men aged 40 (range, 11–70) years, and symptoms endured from 1 month to 10 years.^1^ Lipoma development can be driven by trauma; however, this was not true in our case.^5^

ILs commonly exhibit a lobular or dumbbell-shaped appearance arising from varying tissue pressures during growth. Giant lipomas measure ≥10 cm, and a lipoma should be considered as a differential diagnosis of such large tumours.^6^ There are four cases of giant ILs published in the literature; however, neither exhibited dimensions as substantial as this case.^4^^,^^5^^,^^7^^,^^8^ With gradual growth, the tumour exerts pressure on the blood vessels, lymphatic structures, and crucial nerves.^9^ However, the presence of a mass often serves as the most prevalent subjective symptom in patients with an IL.

Accurate diagnosis and treatment of an IL necessitate the use of appropriate imaging studies, including US, computed tomography (CT), MRI, and histological examination. US has emerged as the preferred imaging modality for lipomas owing to its cost-effectiveness, non-invasiveness, and convenience. The echogenicity of an IL is determined by the uniformity of the mixture comprising adipose and other tissues within the mass. However, relying solely on US data does not yield definitive results. Therefore, the incorporation of CT and MRI is advised for further assessment of the lesion features. On short T1 and long T2 signal MRI, an IL appears as a mass with signal intensity consistent with adipose tissue. Meanwhile, on CT, an IL typically manifests as a homogeneous low attenuation mass. Conversely, malignant liposarcomas demonstrate low to moderate attenuation on imaging, often presenting an inhomogeneous appearance characterised by intermittently disrupted streaks, primarily attributable to cystic regions containing myxoid material. It is important to note that despite negative imaging findings, a liposarcoma can still possess malignant characteristics.^6^ A noteworthy disparity between imaging methods is the visibility of the thin bands with slightly reduced visibility on MRI compared with CT.^10^ Histologically, lipomas display characteristics similar to those of normal adipose tissue. By contrast, well-differentiated liposarcomas exhibit dense collagen bands that traverse the mass, accompanied by gelatinous areas, nuclear pleomorphism, and multinuclear giant cells.^6^^,^^9^

Interventional therapy may be considered unnecessary if an IL does not exhibit symptoms of compression resulting from an excessive volume or a specific anatomical location. In cases of symptom manifestation and vital adjacent organs, tissues, blood vessels, and nerves being subjected to compression, interventional treatment is the primary approach. There are various interventional methods for ILs, including sodium deoxycholate injection, combined steroid and isoproterenol injection, and liposuction. However, surgical resection is the most effective treatment. An IL is characterised by a well-defined pseudocapsule that facilitates surgical dissection.^10^ Excision is recommended in cases meeting the following criteria: tumour size of >5 cm, subfascial location, progressive growth of the tumour, and clinical features such as pain, firmness, or irregularity.^6^ This case satisfied all criteria. Reports on the postoperative IL recurrence rate are varying, ranging from 3% to 62.5%.^1^ However, higher recurrence rates were likely attributable to incomplete surgical excision. Thus, extended and continuous long-term follow-up is crucial for effective monitoring and management of potential recurrences. In cases of recurrence, surgical intervention and radiation are viable treatment options.^9^ In this case, no recurrence was observed.

## Conclusion

Giant ILs between the major and minor pectoral muscles are rare but cause discomfort, pain, and sensory disturbances. US, CT, and MRI are valuable practical tools for diagnostic confirmation. Complete surgical excision followed by histological analysis is the preferred treatment approach, as it not only provides a definitive diagnosis, but also plays a crucial role in minimising the risk of recurrence.

## Funding

This research did not receive any specific grant from funding agencies in the public, commercial, or not-for-profit sectors.

## Conflicts of Interest

None.
